# Development of a diagnostic support system for the fibrosis of nonalcoholic fatty liver disease using artificial intelligence and deep learning

**DOI:** 10.1002/kjm2.12850

**Published:** 2024-05-31

**Authors:** Noppamate Preechathammawong, Mongkon Charoenpitakchai, Nutthawat Wongsason, Julalak Karuehardsuwan, Thaninee Prasoppokakorn, Panyavee Pitisuttithum, Anapat Sanpavat, Karn Yongsiriwit, Thannob Aribarg, Parkpoom Chaisiriprasert, Sombat Treeprasertsuk, Sakkarin Chirapongsathorn

**Affiliations:** ^1^ Division of Gastroenterology and Hepatology, Department of Medicine Phramongkutklao Hospital and College of Medicine Bangkok Thailand; ^2^ Department of Pathology Phramongkutklao College of Medicine Bangkok Thailand; ^3^ Department of Anatomical Pathology Army Institute of Pathology Bangkok Thailand; ^4^ Division of Gastroenterology, Department of Medicine, Faculty of Medicine Chulalongkorn University and King Chulalongkorn Memorial Hospital, Thai Red Cross Society Bangkok Thailand; ^5^ Division of General Internal Medicine, Department of Medicine, Faculty of Medicine Chulalongkorn University and King Chulalongkorn Memorial Hospital, Thai Red Cross Society Bangkok Thailand; ^6^ Department of Pathology, Faculty of Medicine Chulalongkorn University and King Chulalongkorn Memorial Hospital Bangkok Thailand; ^7^ College of Digital Innovation Technology Rangsit University Bangkok Thailand

**Keywords:** artificial intelligence, digital histopathology, liver fibrosis, NAFLD: nonalcoholic fatty liver disease, steatohepatitis

## Abstract

Liver fibrosis is a pathological condition characterized by the abnormal proliferation of liver tissue, subsequently able to progress to cirrhosis or possibly hepatocellular carcinoma. The development of artificial intelligence and deep learning have begun to play a significant role in fibrosis detection. This study aimed to develop SMART AI‐PATHO, a fully automated assessment method combining quantification of histopathological architectural features, to analyze steatosis and fibrosis in nonalcoholic fatty liver disease (NAFLD) core biopsies and employ Metavir fibrosis staging as standard references and fat assessment grading measurement for comparison with the pathologist interpretations. There were 146 participants enrolled in our study. The correlation of Metavir scoring system interpretation between pathologists and SMART AI‐PATHO was significantly correlated (Agreement = 68%, Kappa = 0.59, *p*‐value <0.001), which subgroup analysis of significant fibrosis (Metavir score F2‐F4) and nonsignificant fibrosis (Metavir score F0‐F1) demonstrated substantial correlated results (agreement = 80%, kappa = 0.61, *p*‐value <0.001), corresponding with the correlation of advanced fibrosis (Metavir score F3‐F4) and nonadvanced fibrosis groups (Metavir score F0‐F2), (agreement = 89%, kappa = 0.74, *p*‐value <0.001). SMART AI‐PATHO, the first pivotal artificially intelligent diagnostic tool for the color‐based NAFLD hepatic tissue staging in Thailand, demonstrated satisfactory performance as a pathologist to provide liver fibrosis scoring and steatosis grading. In the future, developing AI algorithms and reliable testing on a larger scale may increase accuracy and contribute to telemedicine consultations for general pathologists in clinical practice.

## 1 INTRODUCTION

Nonalcoholic fatty liver disease (NAFLD) is a hepatic lipid metabolism disease distinguished from alcoholic liver disease by a history of low daily alcohol intake (<210 grams per week in males and < 140 g per week in females for more than 2 years).^1^ NAFLD is usually asymptomatic and can be detected by accident with abdominal ultrasound or radiographic imaging such as computerized tomography (CT scan) or magnetic resonance imaging (MRI). The current global prevalence of NAFLD is estimated to be between 20% and 40%. The prevalence of NAFLD in the Asia‐Pacific area and Thailand are 5%–30% and 30%–34%, respectively.^2^, ^3^, ^4^ According to previous studies, NAFLD is associated with metabolic syndrome and diabetes mellitus. It was detected up to 65% in cardiovascular disease (CVD) and increased the severity and mortality from CVD and circulatory complications.^5^, ^6^, ^7^ In the advanced stage of NAFLD, more than 30% of patients develop cirrhosis and increase the risk for hepatocellular carcinoma (HCC) and other gastrointestinal cancers.

NAFLD has been classified based on clinical presentation into the nonalcoholic fatty liver (NAFL) and nonalcoholic steatohepatitis (NASH). NAFL is a condition of fat deposition in the liver without inflammation. In contrast, NASH is an aggressive form of NAFLD with steatohepatitis. This condition could rapidly lead to cirrhosis and hepatocellular carcinoma.

Liver biopsy is currently the gold standard for diagnosis and is used to assess histology, categorize the severity of the disease, and determine the prognosis.^8^ This procedure is considered an invasive procedure and carries risks of complications such as bleeding, infection, pneumothorax, and pain from the procedure.^9^ NAFLD fibrosis score (NFS) and FIB‐4 index are commonly used for preoperative decision‐making. Contraindications to the procedure must not exist in the patient.^10^, ^11^ To interpret the specimens from this examination, clinicians and pathologists' expertise is essential.

Histological data from liver tissue stained with H&E are currently used to assess and stage hepatic fibrosis.^12^ A pathologist examines the architecture and morphology of the liver to assess the fibrotic stage and grade severity. The Metavir scoring system is widely used to confirm the stage of liver fibrosis.^13^ However, histopathology of liver tissue is based on structural abnormalities associated with fibrosis, which is still a quasi‐quantitative and subjective assessment of the pathologist. Therefore, the assessment of fibrosis may be inaccurate and vary in each center.

Artificial intelligence (AI) and deep learning (DL) also are essential components of the country's social and economic development, especially for the advancement of public health innovations that benefit both patients and medical providers. To improve the efficiency of medical services, AI and information technology must be developed to support the diagnosis, both in terms of reducing diagnostic errors and expanding patient access to telemedicine consultations in rural areas around the country. This study aimed to develop SMART AI‐PATHO, a fully automated assessment method combining quantification of histopathological architectural features, to address unmet needs in the core biopsy evaluation of fibrosis in nonalcoholic NAFLD patients.

## 2 MATERIALS AND METHODS

### 2.1 Study design

This was a cross‐sectional analytic, multicenter observational study, approved by the Institutional Review Board of the Royal Thai Army Medical Department (S060h/63) which was obtained on October 7, 2020, and complied with the Declaration of Helsinki. The study began from October 2020 through October 2022. All NAFLD patients whose diagnosis was confirmed with a liver biopsy in the Division of Gastrointestinal and Hepatology, Department of Medicine, Phramongkutklao Hospital, and Division of Gastroenterology, Department of Medicine, Faculty of Medicine, Chulalongkorn University and King Chulalongkorn Memorial Hospital, Thai Red Cross Society, Bangkok, Thailand, were recruited between October 2015 and October 2021.

### 2.2 Study populations

According to the inclusion criteria, participants had to be at least 18 years old and meet the indication for liver biopsy: intermediate to high risk NAFLD (NFS >−1.455 or FIB‐4 index score >1.45) based on the American Association for the Study of Liver Diseases (AASLD) 2018 diagnostic criteria. The exclusion criteria were alcoholic hepatitis, low‐risk NAFLD, viral hepatitis, HIV, contraindications for liver biopsy, and history of liver cancer patients receiving chemotherapy or radiotherapy.

### 2.3 Procedures

**FIGURE 1:**
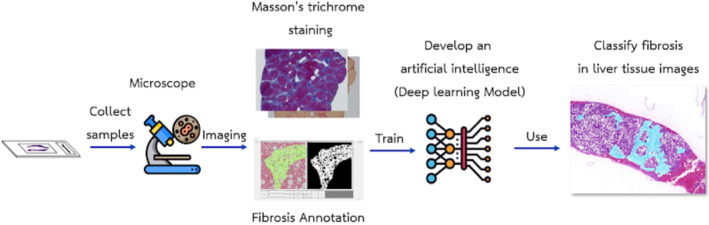
SMART AI‐PATHO online network system.

**FIGURE 2:**
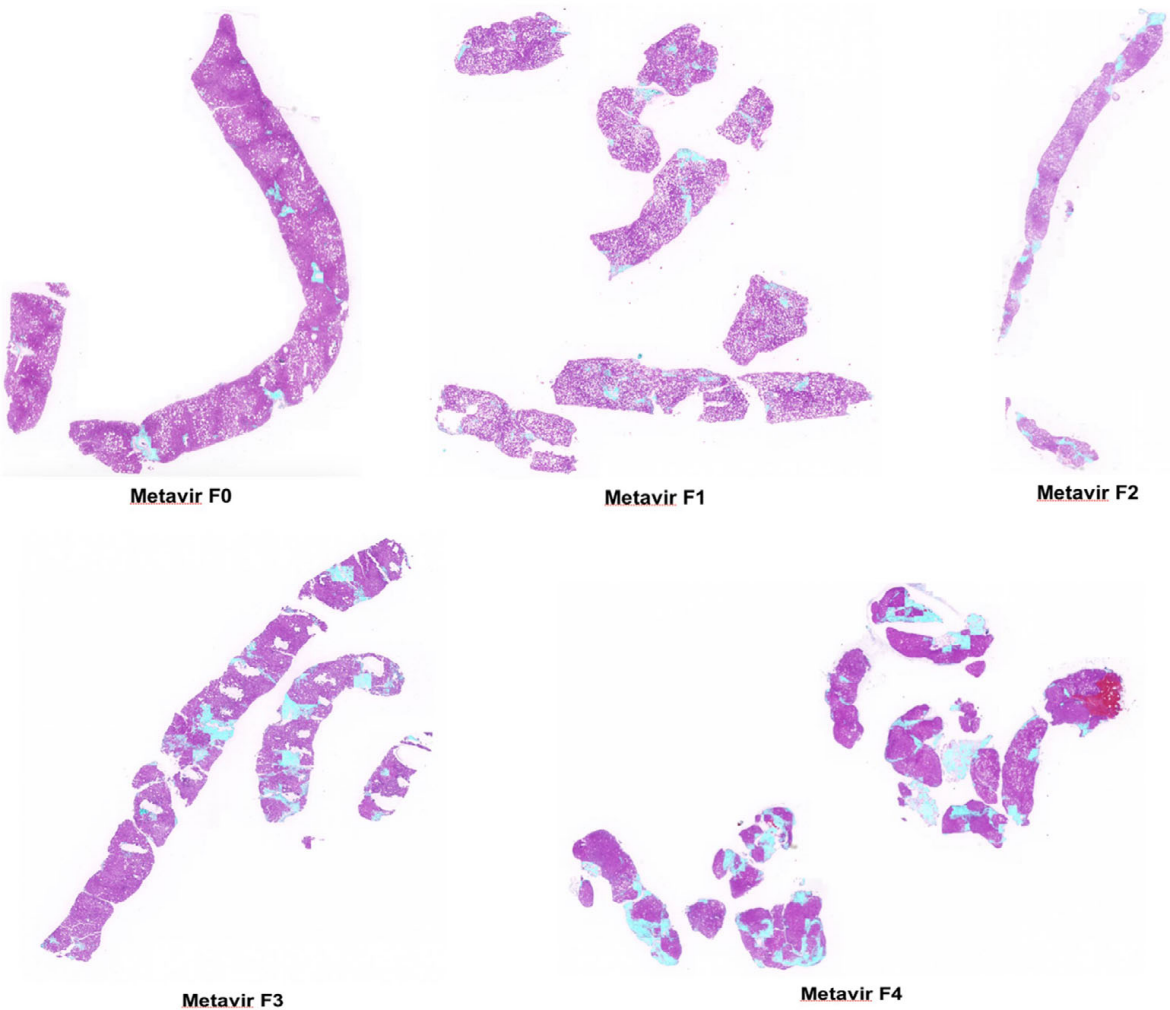
Metavir scoring system: staging of liver fibrosis by SMART AI‐PATHO.

All hepatic tissue specimens were delivered to the Army Institute of Pathology, Phramongkutklao Hospital, or King Chulalongkorn Memorial Hospital for specimens processing once they had been informed about the research and obtained their consent. Cutting specimens from a paraffin block and covering them on glass slides was used to prepare all hepatic tissues. H&E staining was used to stain all hepatic tissue specimens. Previously, a reliability test was performed, 30 hepatic tissue specimens for two pathologists (95% CI 0.98–0.99) which any difference greater than one stage of Metavir staging score was corrected by open adjudication with two pathologist experts. Afterwards, all 146 hepatic tissue specimens were imaged at 4X magnification with whole slide imaging technology (WSI) and processed with AI algorithms to simulate and interpret the stage of fibrosis. The images of the hepatic tissue specimens were in the form of digital photographs (JPEG) which were recorded in the password‐protected file using the coding system to define the code of the digital images and they were transferred via an online network system (Figure 1). The AI algorithm's reliability was also tested to interpret the stage of fibrosis before the actual examination began. Likewise, hepatic tissue specimens were interpreted by pathologists for assessing the severity of disease and staging of fibrosis (Metavir scoring system, Figure 2). Baseline characteristics such as age, gender, weight, height, the body mass index (BMI), waist circumference, blood pressure, underlying disease, current medication, and laboratory data, were obtained from electronic medical records.

### 2.4 AI and DL

**FIGURE 3:**
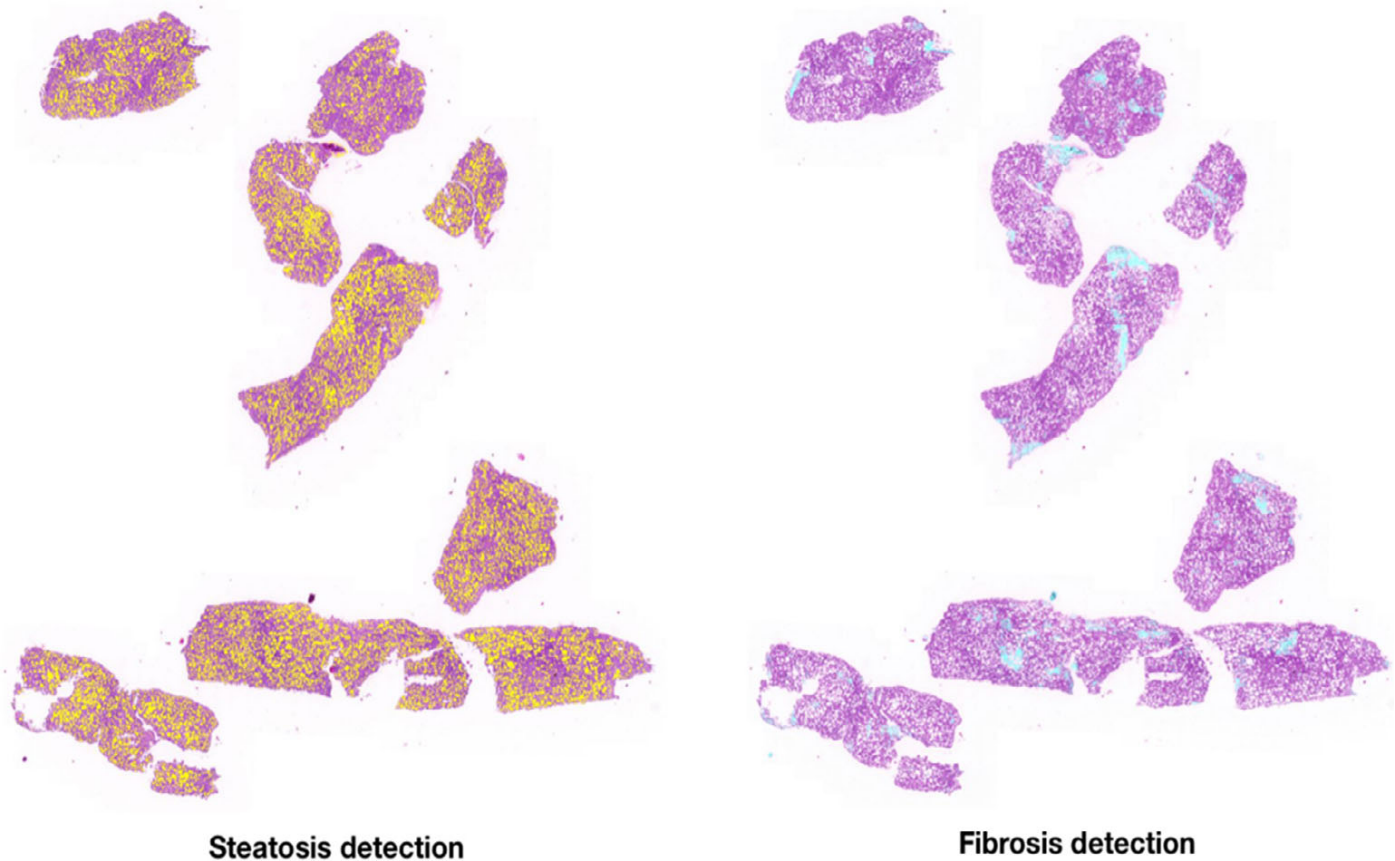
Annotated area of fibrosis and steatosis by SMART AI‐PATHO.

SMART AI‐PATHO, artificially intelligent algorithms were developed by the College of Digital Innovation Technology, Rangsit University, Pathum Thani, Thailand, to detect an area of fibrosis and steatosis in hepatic tissue images (Figure 3). More details are provided in the Supplementary Information.

### 2.5 Outcomes

The primary outcome was a correlation of hepatic fibrosis interpretation between pathologists and AI algorithms (SMART AI‐PATHO) in NAFLD patients. Secondary outcomes were evaluated through subgroup analysis of the correlation between disease severity and fibrosis stages in NAFLD patients.

### 2.6 Sample size and statistical analyses

The sample size in this study was 146. The Kolmogorov–Smirnov test was used to verify the normal assumption of quantitative data. For continuous data were present in as mean ± standard deviation. For binary and categorical data were shown as frequency, proportion, and percentage, and categorical data was compared with the chi‐square test. The intra‐class correlation coefficient was used to report the pathologists’ and SMART AI‐PATHO's reliability. Fibrosis stage interpretation between pathologists’ and SMART AI‐PATHO in NAFLD patients was reported with a kappa coefficient statistic (*k*‐value). The 95% confidential interval was calculated and a *p*‐value <0.05 was considered statistically significant.

## 3 RESULTS

All 146 participants were included in this study. Baseline characteristics, such as the gender, age, BMI, underlying disease, current medication, and laboratory data, were reported in Table 1. The average age of the study population was 47 ± 15 years and included more women (58%) than men (42%). The BMI was 39.5 ± 15.2 kg/m^2^. Diabetes mellitus, hypertension, and hyperlipidemia were reported as an underlying disease about 29%, 46%, and 45%, consecutively.

**TABLE 1:** Baseline characteristics (*n* = 146).

	Test (*n* = 146)	Validity (30)	*p*‐Value
Gender			
Male	61 (41.8%)	14 (46.7%)	0.622
Female	85 (58.2%)	16 (53.3%)	
Age (years)	47.24 ± 15.17	54.53 ± 16.61	**0.019**
Height (cm)	163.93 ± 9.82	163.07 ± 10.15	0.663
Weight (kg)	107.55 ± 47.28	91.37 ± 47	0.089
BMI (kg/m^2^)	39.5 ± 15.23	33.44 ± 14.61	0.047
<25	28 (19.2%)	8 (26.7%)	0.354
≥25	118 (80.9%)	22 (73.3%)	
Underlying disease			
Diabetes mellitus	43 (29.5%)	5 (16.7%)	0.152
Hypertension	68 (46.6%)	8 (26.7%)	**0.045**
Hyperlipidemia	66 (45.2%)	9 (30%)	0.125
Cardiovascular disease	5 (3.4%)	2 (6.7%)	0.408
Medication			
Aspirin	9 (6.2%)	3 (10%)	0.448
Metformin	35 (24%)	4 (13.3%)	0.201
Thiazolidinedione	1 (0.7%)	0 (0%)	0.649
Statin	47 (32.2%)	8 (26.7%)	0.552
ACEI/ ARB	31 (21.2%)	2 (6.7%)	0.063
Laboratory			
Hb (g/dL)	13.59 ± 3.22	13.12 ± 1.38	0.543
WBC (cells/ul)	8291.54 ± 2355.82	6837.78 ± 1406.32	**0.001**
Creatinine (mg/dL)	0.71 ± 0.18	0.76 ± 0.21	0.363
Albumin (g/dL)	4.19 ± 0.37	4.18 ± 0.35	0.903
Globulin (g/dL)	3.46 ± 0.71	3.55 ± 0.83	0.544
Total bilirubin (mg/dL)	0.67 ± 0.64	0.75 ± 0.7	0.627
AST (U/L)	61.42 ± 60.72	62.52 ± 39.67	0.928
ALT (U/L)	74.8 ± 59.8	81.41 ± 56.65	0.596
ALP (U/L)	82.65 ± 34.63	88.78 ± 29.74	0.478
Cholesterol (mg/dL)	190.78 ± 46.45	200.52 ± 57.57	0.331
Triglyceride (mg/dL)	157.02 ± 91.38	159.34 ± 116.3	0.906
HDL (mg/dL)	56.72 ± 75.12	84.46 ± 153.73	0.359
LDL (mg/dL)	120.58 ± 37.79	125 ± 35.24	0.585
FPG (mg/dL)	113.78 ± 30.67	112.4 ± 26.34	0.820
HbA1C (%)	6.34 ± 1.29	6.12 ± 0.96	0.401

The pathologist's interpretation results were reported in Table 2. The steatosis percentage was 23.39 ± 18.15%. The NASH activity score was 2.81 ± 1.33. Metavir scores F0–F4 were 27.4%, 25.3%, 21.9%, 17.1% and 8.2%, respectively.

**TABLE 2:** Hepatic tissue pathological interpretation.

	Steatosis	Lobular inflammation	Hepatocyte ballooning	Metavir scoring system
0	7 (4.8%)	50 (34.2%)	41 (28.1%)	40 (27.4%)
1	117 (80.1%)	75 (51.4%)	85 (58.2%)	37 (25.3%)
2	13 (8.9%)	20 (13.7%)	20 (13.7%)	32 (21.9%)
3	9 (6.2%)	1 (0.7%)		25 (17.1%)
4				12 (8.2%)
**Steatosis (%)** 23.39 ± 18.15 (0–90)				
**NAFLD activity score** 2.81 ± 1.33 (0–7)				

Meanwhile, the percentage of fibrosis from SMART AI‐PATHO was 2.18 ± 5.74%. The steatosis percentage was 11.5 ± 6.68%. Metavir scores F1‐F4 were 30.8%, 21.9%, 18.5%, 20.5% and, 8.2%, as demonstrated in Table 3.

**TABLE 3:** SMART AI‐PATHO Interpretation.

	*N* (%) or mean ± SD.	Min–max
Fibrosis (%)	2.18 ± 5.74	0–41
Steatosis (%)	11.5 ± 6.68	0.85–38.36
Metavir scoring system		
0	45 (30.8%)	
1	32 (21.9%)	
2	27 (18.5%)	
3	30 (20.5%)	
4	12 (8.2%)	

For the primary outcome (Table 4), The inter‐rater reliability of Metavir scoring system interpretation between pathologists and SMART AI‐PATHO was average agreement at 68.4%, and the Kappa value was 0.59, *p*‐value <0.001. Subgroup analysis of significant fibrosis (Metavir score F2–F4) and nonsignificant fibrosis (Metavir score F0–F1) demonstrated substantial correlated results (agreement = 80.8%, kappa = 0.61, *p*‐value <0.001, Table 5). Same as the correlation of advanced fibrosis (Metavir score F3–F4) and nonadvanced fibrosis (Metavir score F0–F2), (agreement = 89.7%, kappa = 0.74, *p*‐value <0.001, Table 6).

**TABLE 4:** Correlation of metavir scoring system interpretation between pathologists and SMART AI‐PATHO.

Metavir scoring system	Pathologist^a^					
	0	1	2	3	4	Total
AI^b^						
0	30	7	6	2	0	45
1	5	21	4	1	1	32
2	2	4	20	0	1	27
3	2	4	1	21	2	30
4	1	1	1	1	8	12
Total	40	37	32	25	12	146

*Note*: Agreement = 68.49%, kappa = 0.5948, and *p*‐value <0.001.

^a^ Metavir scoring system interpreted by pathologist.

^b^ Metavir scoring system annotated by SMART AI PATHO.

**TABLE 5:** Subgroup analysis of the correlation of significant fibrosis of Metavir scoring system interpretation between pathologists and SMART AI‐PATHO.

Metavir scoring system: Significant fibrosis subgroup	Pathologist*		
	0–1	2–4	Total
AI**			
0–1	63	14	77
2–4	14	55	69
Total	77	69	146

*Note*: Agreement = 80.82%, kappa = 0.6153, and *p*‐value <0.001.

**TABLE 6:** Subgroup analysis of the correlation of advanced fibrosis of Metavir scoring system interpretation between pathologists and SMART AI‐PATHO.

Metavir scoring system: Advanced fibrosis subgroup	Pathologist*		
	0–2	3–4	Total
AI**			
0–2	99	5	104
3–4	10	32	42
Total	109	37	146

*Note*: Agreement = 89.73%, kappa = 0.7401, and *p*‐value <0.001.

In the secondary outcome analysis, Metavir staging from AI has a statistically significant positive correlation with FIB‐4 risk index (*p‐*value <0.001) even subgroup into significant fibrosis (Metavir score F2–F4) and nonsignificant fibrosis group (Metavir score F0–F1) (*p‐*value = 0.028), as shown in Table 7.

**TABLE 7:** Correlation between Metavir staging from AI algorithms and FIB‐4 risk index.

AI Metavir stage	FIB‐4 score risk index				*p‐*Value
	Low	Moderate	High	Total	
0	31	4	2	37	**<0.001** *
1	11	11	2	24	
2	16	9	0	25	
3	8	10	5	23	
4	1	4	3	8	
Nonsignificant fibrosis					
F0‐1	42	15	4	61	**0.028** *
Significant fibrosis					
F2‐4	25	23	8	56	
Total	67	38	12	117	

* *Chi‐square test*.

The clinical and laboratory profile association aspect, the BMI was found that inversely correlated with Metavir score system especially in an overweight BMI subgroup (≥25 kg/m^2^) (pathologist: *r* = −0.276, *p*‐value = 0.003), (AI: *r* = −0.292, *p*‐value <0.001). The white blood count (WBC) was also inversely correlated with percentage of fibrosis annotated by SMART AI‐PATHO (*r* = −0.261, *p*‐value = 0.005). Serum Globulin and Total bilirubin (TB) were statistically significant correlated with Pathologist Metavir staging (Globulin: *r* = 0.204, *p*‐value = 0.016, TB: *r* = 0.197, *p*‐value <0.033). The clinical and laboratory data analyses are reported in Table 8.

**TABLE 8:** Clinical‐laboratory data and hepatopathological interpretation correlation.

	Metavir stage				% fibrosis		% steatosis			
	Pathologist		AI		AI		Pathologist		AI	
	*r*	*p*	*r*	*p*	*r*	*p*	*r*	*p*	*r*	*p*
BMI (kg/m^2^)	**−0.169**	**0.042**	**−0.292**	**<0.001**	**−0.521**	**<0.001**	−0.15	0.071	0.019	0.821
BMI <25	0.318	0.106	0.24	0.228	0.118	0.557	−0.043	0.83	0.158	0.432
BMI ≥25	**−0.276**	**0.003**	**−0.388**	**<0.001**	**−0.523**	**<0.001**	**−0.224**	**0.015**	−0.09	0.332
Hb	0.061	0.514	−0.038	0.681	−0.177	0.056	0.036	0.7	−0.007	0.941
WBC	−0.063	0.497	−0.133	0.152	**−0.261**	**0.005**	−0.012	0.897	0.127	0.174
Cr	−0.004	0.968	0.066	0.479	0.178	0.055	0.046	0.624	0.025	0.788
Alb	−0.057	0.493	−0.018	0.83	0.064	0.442	0.061	0.463	0.097	0.244
Globulin	**0.204**	**0.016**	0.105	0.217	−0.056	0.516	−0.088	0.304	−0.146	0.086
TB	**0.197**	**0.033**	0.117	0.21	−0.076	0.414	−0.005	0.96	0.1	0.285
AST	**0.407**	**<0.001**	**0.343**	**<0.001**	**0.382**	**<0.001**	**0.208**	**0.013**	0.073	0.387
ALT	**0.31**	**<0.001**	**0.255**	**0.002**	**0.343**	**<0.001**	**0.228**	**0.006**	0.058	0.489
ALP	0.119	0.202	0.049	0.602	0.072	0.437	−0.095	0.308	−0.071	0.444
Chol.	−0.132	0.136	−0.138	0.117	−0.083	0.349	−0.075	0.397	−0.023	0.794
TG	−0.016	0.859	−0.002	0.982	0.112	0.206	0.138	0.118	0.132	0.133
HDL	0.048	0.593	0.057	0.525	0.089	0.321	−0.098	0.271	−0.108	0.227
LDL	−0.121	0.225	−0.062	0.531	−0.09	0.368	−0.032	0.751	0.026	0.792
FBS	**0.306**	**<0.001**	**0.221**	**0.01**	0.154	0.073	**0.237**	**0.005**	0.123	0.155
HbA1C	**0.246**	**0.005**	0.093	0.299	−0.005	0.952	0.112	0.21	0.069	0.444

## 4 DISCUSSION

This is Thailand's first innovative medical scientific study to analyze the agreement between pathologists and AI algorithm in detecting fibrosis in NAFLD patients. The results reveal a prominent level of agreement in detecting liver fibrosis and Metavir staging. Agreements in detecting liver fibrosis were higher when categorized into significant fibrosis group (F2–F4) and nonsignificant fibrosis group (F0–F1). Agreement in detecting liver fibrosis was the highest when categorized into advanced fibrosis group (F3–F4) and nonadvanced fibrosis group (F0–F2) as previous study.^14^ From this study we think SMART AI PATHO can detect significant fibrosis stage as well as detection and interpretation by pathologist. In comparison to the previous study,^15^ which focused on the AI detection of liver fibrosis in hepatitis B patients, there was an excellent consensus value (AUC 0.84–0.97) greater than our study. This may be because our subjects had more variety of co‐morbidity that could affect steatohepatitis than focus on only hepatitis B patients. In this study, higher FIB‐4 scores were associated with more Metavir stages, which is consistent with current knowledge.^16^, ^17^

Regarding the agreement, the pathologists and SMART AI‐PATHO's assessments of liver fibrosis were highly comparable. However, in this study, it was discovered that participants with BMI ≥25 kg/m^2^ revealed an inverse association with the degree of liver fibrosis, which has been reported in some previous research.^18^, ^19^ In the author's opinion, further studies should be performed on a more extensive and varied sample size to obtain clinical advantages. AST, ALT, and total bilirubin levels were favorably correlated with both pathologist and SMART AI‐PATHO Metavir stage, AI hepatosteatosis, and fibrosis percentage, similar to previous research.^18^, ^20^ According to the pathology of NAFLD, this condition causes a chronic inflammatory process and progresses to a fibrotic state, associated with a higher degree of fibrosis and steatosis as well. Hepatosteatosis percentage and Metavir stage (as determined by SMART AI‐PATHO and pathologist assessments) were also correlated with FBS and HbA1C levels,^18^, ^21^ as it is known that diabetes is considered a risk factor for NAFLD.^22^

Although there was a substantial agreement between AI and pathologists interpretation for the primary outcome. The secondary outcomes of this study demonstrated that clinical variables and the hepatosteatosis/liver fibrosis revealed a statistically significant in slightly positive correlation, which the degree of relationship among the variables depends on the results of previous research. A review of prior research reveals that no comparison with AI has ever been established. The authors suggest considering for the concordant direction of the results of further study.

This study also had some limitations. While SMART AI‐PATHO can analyze the percentage area of hepatic fibrosis and steatosis. This AI algorithm has limitations in assessing lobular inflammation, hepatocyte ballooning, or even pattern of fibrosis based on the Metavir grading system. Consequently, it is considered that although AI may assist in initial assessment of NAFLD, it cannot completely substitute pathologist interpretation. Future AI software that evaluates disease activity may also be developed. Improving the ability to evaluate other various specimen staining techniques should be considered in future studies as well.

Second, limitations and missing data from the retrospective study make it challenging to fully examine some statistical data. The quality of the specimen slides must then be examined. The size of the biopsy, dyeing techniques, the fade of the specimen slide, and other specimen‐related factors all have an impact on the interpretation's outcomes. The authors advise calibrating the color grading range before the analysis to standardize the evaluation. Third, this study could not identify inflammatory cells and features of steatohepatitis by our AI.

In conclusion, the SMART AI‐PATHO is a modern innovative technology that could assist pathologists in accuracy of liver fibrosis detection whether eliminating pathologist misdiagnosis and/or interpretation bias. Furthermore, it is also beneficial in terms of telemedicine consultations and can be developed more to identify other histology of steatohepatitis in NAFLD.

## CONFLICT OF INTEREST STATEMENT

All authors declare that there are no conflicts of interest.

## ETHICS STATEMENT

All subjects were properly instructed and consented to participate in this trial by signing the informed consent regulation provided by the Institutional Review Board of the Royal Thai Army Medical Department Committee (IRB number S060h/63). The Institutional Review Board of the Royal Thai Army Medical Department Committee uses the World Medical Association: DECLARATION OF HELSINKI, GUIDELINES FOR GOOD CLINICAL PRACTICE: ICH Harmonized Tripartite Guideline, Council for International Organizations of Medical Sciences (CIOMS), CODE of FEDERAL REGULATIONS: Title 45 Public Welfare; Part 46 Protection of Human Subjects and the Belmont Report to regulate the ethics concerns in publications. Informed consent was obtained from all subjects, and all methods were conducted according to the relevant guidelines and regulations.

## Supporting information


**Data S1.** Supporting Information.

## References

[1] Rinella ME , Neuschwander‐Tetri BA , Siddiqui MS , Abdelmalek MF , Caldwell S , Barb D , et al. AASLD practice guidance on the clinical assessment and management of nonalcoholic fatty liver disease. Hepatology. 2023;77(5):1797–1835. 36727674 10.1097/HEP.0000000000000323 PMC10735173

[2] Chitturi S , Farrell GC , Hashimoto E , Saibara T , Lau GK , Sollano JD , et al. Non‐alcoholic fatty liver disease in the Asia‐Pacific region: definitions and overview of proposed guidelines. J Gastroenterol Hepatol. 2007;22(6):778–787. 17565630 10.1111/j.1440-1746.2007.05001.x

[3] Kladchareon N , Treeprasertsuk S , Mahachai V , Wilairatana P , Kullavanijaya P . The prevalence of nonalcoholic steatohepatitis in Thai patients with non‐HBV, non‐HCV chronic hepatitis. J Med Assoc Thail. 2004;87(Suppl 2):S29–S34. 16083157

[4] Younossi Z , Anstee QM , Marietti M , Hardy T , Henry L , Eslam M , et al. Global burden of NAFLD and NASH: trends, predictions, risk factors and prevention. Nat Rev Gastroenterol Hepatol. 2018;15(1):11–20. 28930295 10.1038/nrgastro.2017.109

[5] Gupte P , Amarapurkar D , Agal S , Baijal R , Kulshrestha P , Pramanik S , et al. Non‐alcoholic steatohepatitis in type 2 diabetes mellitus. J Gastroenterol Hepatol. 2004;19(8):854–858. 15242486 10.1111/j.1440-1746.2004.03312.x

[6] Targher G , Bertolini L , Rodella S , Tessari R , Zenari L , Lippi G , et al. Nonalcoholic fatty liver disease is independently associated with an increased incidence of cardiovascular events in type 2 diabetic patients. Diabetes Care. 2007;30(8):2119–2121. 17519430 10.2337/dc07-0349

[7] Younossi ZM , Golabi P , de Avila L , Paik JM , Srishord M , Fukui N , et al. The global epidemiology of NAFLD and NASH in patients with type 2 diabetes: a systematic review and meta‐analysis. J Hepatol. 2019;71(4):793–801. 31279902 10.1016/j.jhep.2019.06.021

[8] Berger D , Desai V , Janardhan S . Con: liver biopsy remains the gold standard to evaluate fibrosis in patients with nonalcoholic fatty liver disease. Clin Liver Dis (Hoboken). 2019;13(4):114–116. 31061705 10.1002/cld.740 PMC6491029

[9] Thampanitchawong P , Piratvisuth T . Liver biopsy:complications and risk factors. World J Gastroenterol. 1999;5(4):301–304. 11819452 10.3748/wjg.v5.i4.301 PMC4695539

[10] Salomone F , Micek A , Godos J . Simple scores of fibrosis and mortality in patients with NAFLD: a systematic review with meta‐analysis. J Clin Med. 2018;7(8):219. 30111756 10.3390/jcm7080219 PMC6111765

[11] Sterling RK , Lissen E , Clumeck N , Sola R , Correa MC , Montaner J , et al. Development of a simple noninvasive index to predict significant fibrosis in patients with HIV/HCV coinfection. Hepatology. 2006;43(6):1317–1325. 16729309 10.1002/hep.21178

[12] Lefkowitch JH . Special stains in diagnostic liver pathology. Semin Diagn Pathol. 2006;23(3–4):190–198. 17355092 10.1053/j.semdp.2006.11.006

[13] Bedossa P . Presentation of a grid for computer analysis for compilation of histopathologic lesions in chronic viral hepatitis C. Cooperative study of the METAVIR group. Ann Pathol. 1993;13:260–265. 8280302

[14] Okanoue T , Shima T , Mitsumoto Y , Umemura A , Yamaguchi K , Itoh Y , et al. Novel artificial intelligent/neural network system for staging of nonalcoholic steatohepatitis. Hepatol Res. 2021;51(10):1044–1057. 34124830 10.1111/hepr.13681

[15] Xu S , Wang Y , Tai DCS , Wang S , Cheng CL , Peng Q , et al. qFibrosis: a fully‐quantitative innovative method incorporating histological features to facilitate accurate fibrosis scoring in animal model and chronic hepatitis B patients. J Hepatol. 2014;61(2):260–269. 24583249 10.1016/j.jhep.2014.02.015 PMC4278959

[16] Xu XL , Jiang LS , Wu CS , Pan LY , Lou ZQ , Peng CT , et al. The role of fibrosis index FIB‐4 in predicting liver fibrosis stage and clinical prognosis: a diagnostic or screening tool? J Formos Med Assoc. 2022;121(2):454–466. 34325952 10.1016/j.jfma.2021.07.013

[17] Rungta S , Kumari S , Deep A , Verma K , Swaroop S . APRI and FIB‐4 performance to assess liver fibrosis against predefined Fibroscan values in chronic hepatitis C virus infection. J Family Med Prim Care. 2021;10(11):4082–4088. 35136771 10.4103/jfmpc.jfmpc_666_21 PMC8797084

[18] Nah EH , Cho S , Kim S , Chu J , Kwon E , Cho HI . Prevalence of liver fibrosis and associated risk factors in the Korean general population: a retrospective cross‐sectional study. BMJ Open. 2021;11(3):e046529. 10.1136/bmjopen-2020-046529 PMC7993338 33762246

[19] Gopalakrishna H , Fashanu OE , Nair GB , Ravendhran N . Association between body mass index and liver stiffness measurement using transient elastography in patients with non‐alcoholic fatty liver disease in a hepatology clinic: a cross sectional study. Transl Gastroenterol Hepatol. 2023;8:10. 36704647 10.21037/tgh-22-1 PMC9813648

[20] Zou Y , Zhong L , Hu C , Sheng G . Association between the alanine aminotransferase/aspartate aminotransferase ratio and new‐onset non‐alcoholic fatty liver disease in a nonobese Chinese population: a population‐based longitudinal study. Lipids Health Dis. 2020;19(1):245. 33239040 10.1186/s12944-020-01419-z PMC7690093

[21] Mukherjee S , Saha S , Banerjee U , Banerjee AK , Banerjee R . A correlational study of hepatic steatosis (fatty liver disease) and liver enzymes (ALT, AST, GGT) in the scenario of insulin resistance among young medicos. Biosci Biotechnol Res Asia. 2020;17(4):717–725.

[22] Tomah S , Alkhouri N , Hamdy O . Nonalcoholic fatty liver disease and type 2 diabetes: where do diabetologists stand? Clin Diabetes Endocrinol. 2020;6:9. 32518675 10.1186/s40842-020-00097-1 PMC7275502

