# Supplementary material for: Development of a diagnostic support system for the fibrosis of nonalcoholic fatty liver disease using artificial intelligence and deep learning
Source: Kaohsiung J Med Sci. 2024 May 31;40(8):757–65. doi: 10.1002/kjm2.12850 (PMC11895648; doi:10.1002/kjm2.12850)
Supplement: Supplementary file 1 — Data S1. Supporting Information. [file KJM2-40-757-s001.docx]

**Supplementary**

**AI-PATHO model development**

This research project has developed two models: a model for predicting the quantity of fat appearing on liver tissue and a model for predicting the location and shape of fibrosis appearing on liver tissue. The details are as follows:

**The model for predicting the quantity of fat appearing on liver tissue**

The process of developing the model for predicting the quantity of fat appearing on liver tissue using image processing, as illustrated in the following figure 1.


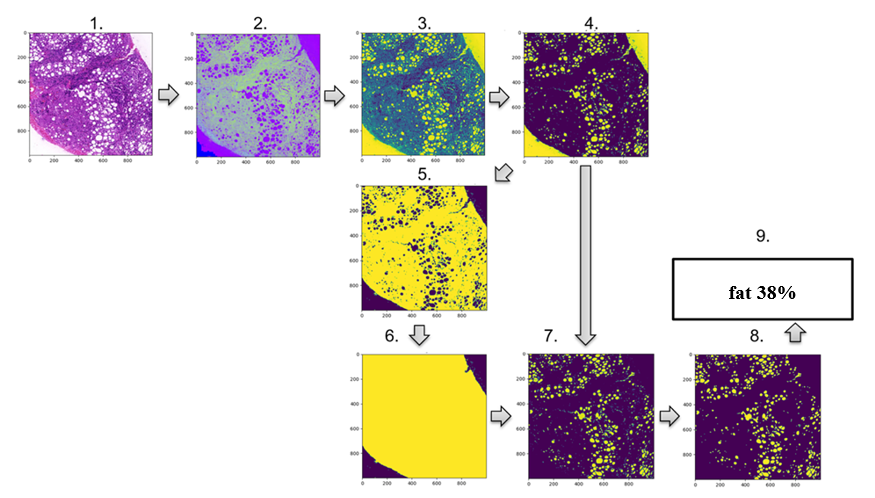


The steps involved in developing the model for predicting the quantity of fat present on liver tissue using image processing.

From the figure, the details of the operations in each step (according to the numbers within the figure) are as follows:

1. Step 1: The image depicts liver cell tissue stained and stored in the RGB color space, which is a combination of red (R), green (G), and blue (B) colors.
2. The RGB color space is converted to the HSV color space, which consists of the Hue, Saturation, and Value components.
3. The colored image is transformed into a grayscale image, where the intensity levels of the colors are reduced to a range between white and black, and any grayscale level below 210 is set to 0 (black).
4. In the grayscale image, pixels with HSV values satisfying the condition S > 10 and V < 240 are set to 0 (black).
5. The grayscale image is further processed using Otsu’s Binarization technique, which determines the threshold value (T) that minimizes the within-class variance (σ_within^2) of both classes separated by T. This technique involves finding T using the following equation:

$$\sigma_{within}^{2}\left( T \right)=n_{1}\left( T \right)\sigma_{1}^{2}\left( T \right)+n_{2}\left( T \right)\sigma_{2}^{2}\left( T \right)$$

where $n_{1}\left( T \right)=\left| \left\{ I\left( x,y \right)<T \right\} \right|,n_{2}\left( T \right)=|I(x,y)\geq T|$

1. Filling color into the areas that are void will result in an image representing the entire liver cell area.
2. Involves extracting only the liver cell area from the image labeled as number 6.
3. Entails removing noise from the image using Smoothing noise technique, resulting in an image representing the fat area.
4. Combines the entire liver cell area image (number 6) and the fat area image (number 8) to calculate the quantity of fat. This is represented as a percentage of fat content, as illustrated in the example in the result image showing a fat quantity of 38%.

**The model for predicting the position and shape of fibrosis nodules appearing on liver tissue.**

This research project involves a step-by-step process in developing a model for classifying the position and shape of fibrosis nodules appearing on liver tissue. The sequence of steps in developing the model is illustrated in the following figure 2.

**
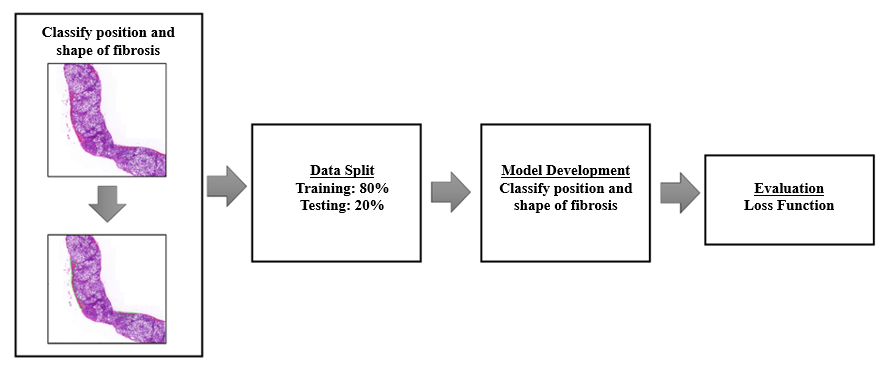
**

The steps involved in developing the model to classify the position and shape of fibrosis nodules appearing on liver tissue.

From the above figure, the details of the operations in each step are as follows:

- Classify position and shape of fibrosis: This step involves classifying the position and shape of fibrosis nodules on liver tissue by expert physicians specializing in fibrosis. Tools in section 2.6 are utilized. Subsequently, all images are gathered, including actual image portions segmented (Figure 3), to serve as input data and images resulting from description classification (Figure 4) to serve as output data for model training.


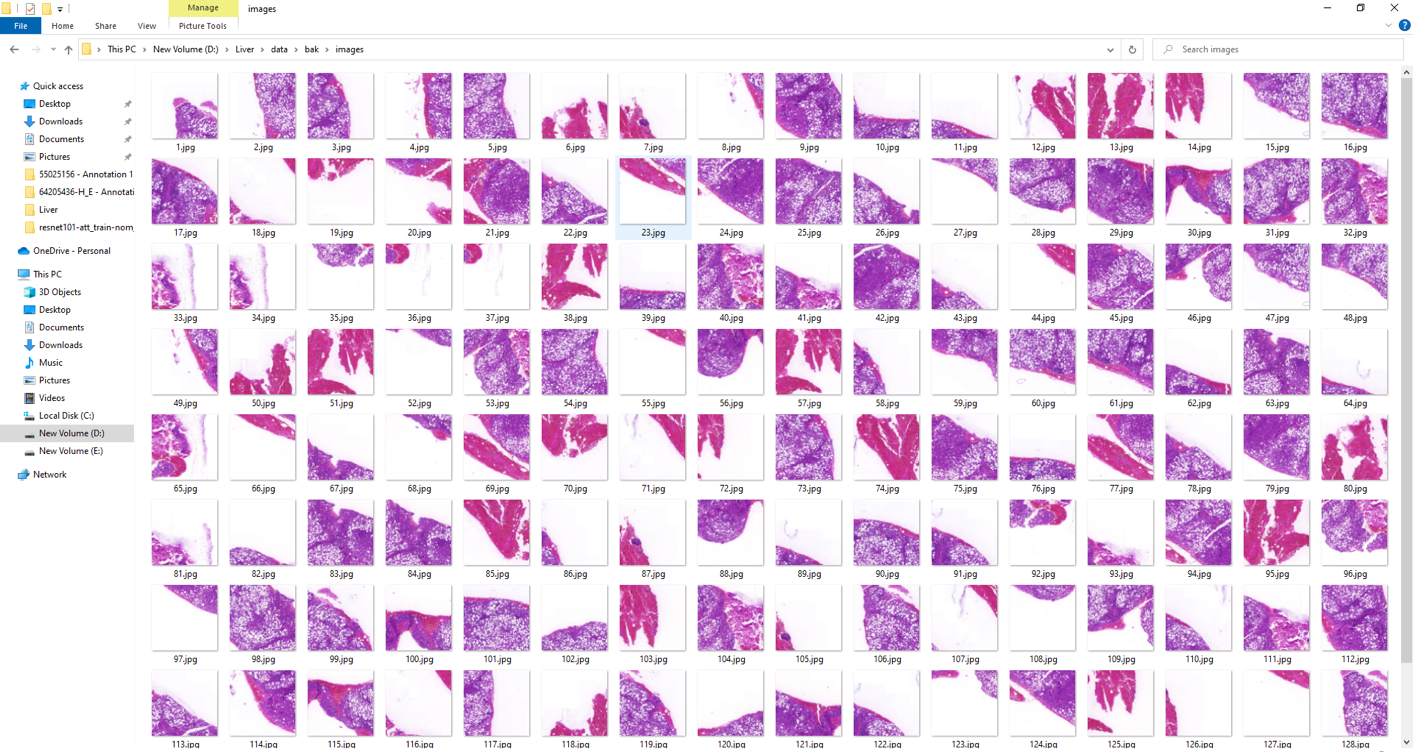


**Figure 3**. Gathering actual images segmented into parts to be used as input data for model training


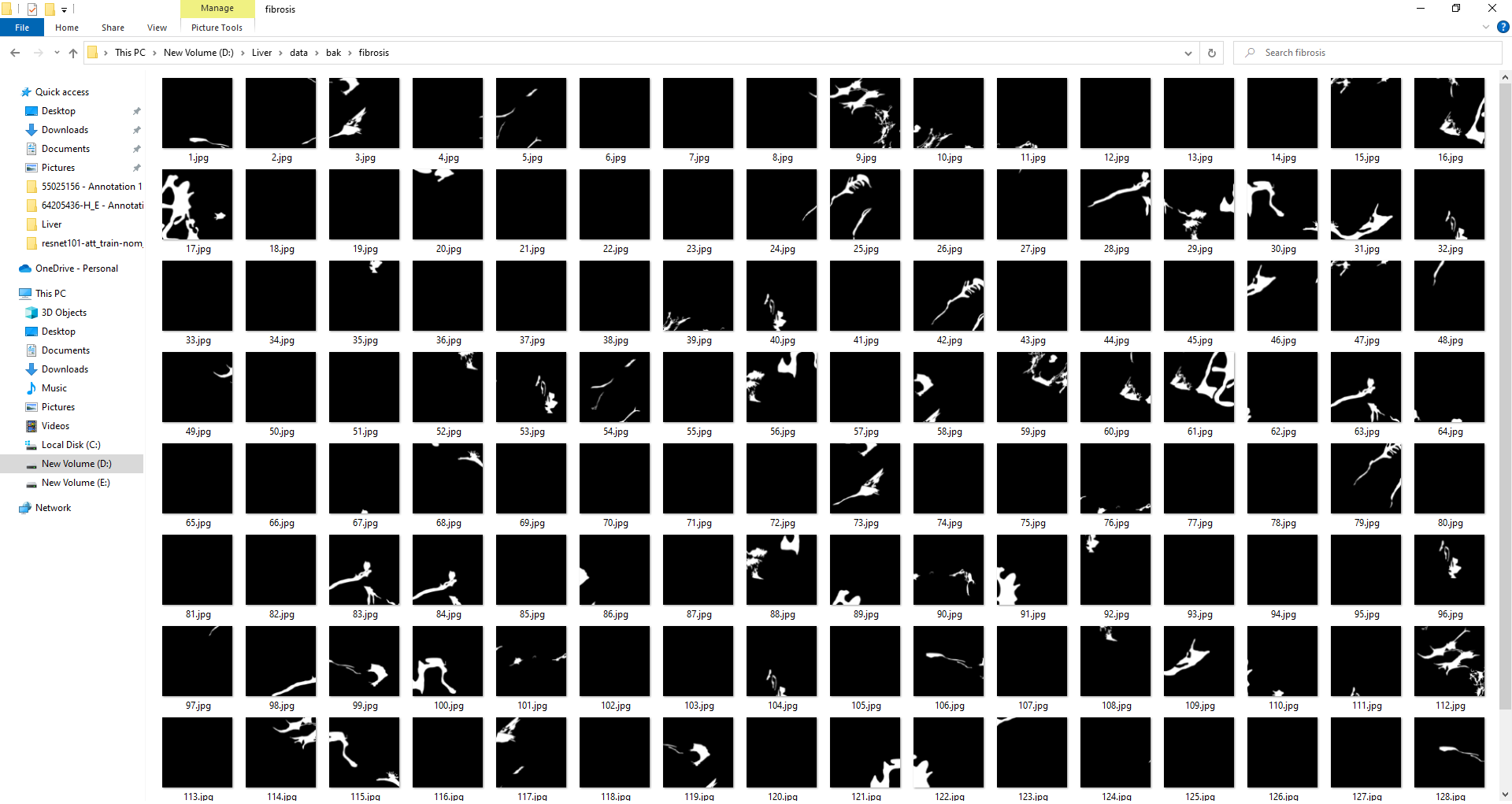


**Figure 4**. Gathering images resulting from annotation for use as input data for model training.

- Data Splitting: This research divides the data into two sets: a training dataset, comprising 80% of the total data (1,752 images), and a testing dataset, comprising 20% of the total data (438 images).
- Model Development: The model for identifying the position and shape of fibrosis nodules was developed using the U-Net model, which is a Convolutional Neural Network (CNN) architecture commonly used for image segmentation tasks. This research project applies it to the task of identifying the position and shape of fibrosis nodules. The U-Net architecture is structured as illustrated in the following figure.


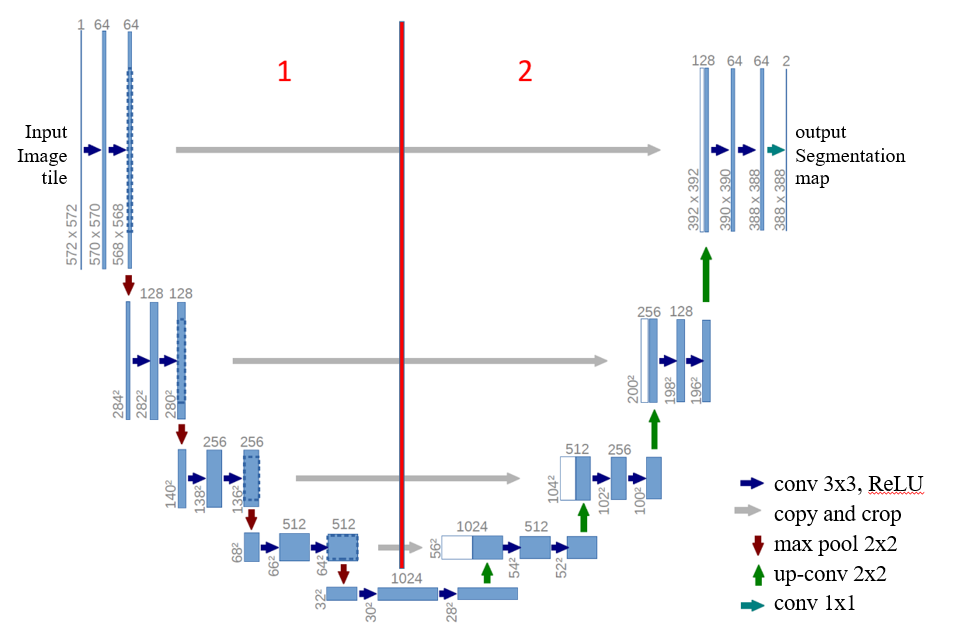


**Figure 5.** U-Net Structure

From the above figure, it can be observed that the structure of the U-Net is divided into two parts: *Part 1 (left side)*: Comprises convolutional layers of size 3x3 (conv 3x3) and performs down-sampling using Max Pooling (max pool 2x2) to extract features from the input image. This process involves extracting features from high-resolution to low-resolution levels. *Part 2 (right side)*: Involves up-sampling (up-conv 2x2) followed by convolutional layers, utilizing the features obtained from Part 1 to generate the segmented output image. The final layer provides the output with two classes: liver tissue area and fibrosis nodules. An example of the segmented fibrosis nodules classification result is depicted in the following figure.


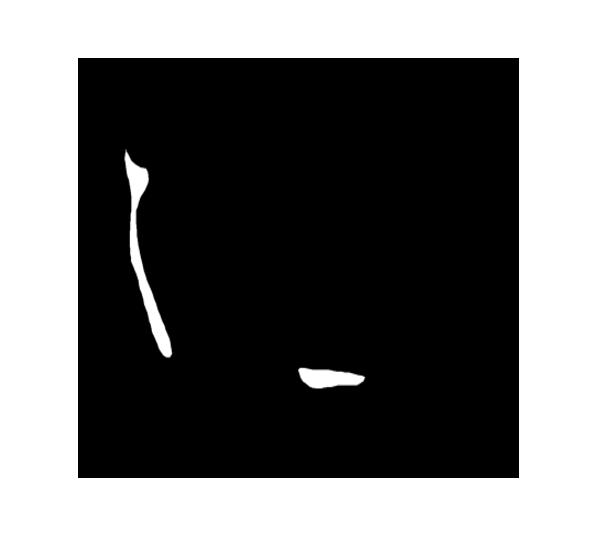


An example of the result of fibrosis nodules classification

The research team utilized the U-Net architecture combined with the application of ResNet18, ResNet34, and ResNet101 architectures, which were designed in three variations. The details are as follows:

**Table 1.** Architecture structure

| layer name | output size | 18-layer | 34-layer | 101-layer |
| --- | --- | --- | --- | --- |
| conv1 | 256x256 | 16x16, 64, stride 2 | | |
| conv2_x | 128x128 | 3x3 max pool, stride 2 | | |
|  |  | $\left[ \begin{matrix} 3\times3, 64 \\ 3\times3, 64 \end{matrix} \right]\times2$ | $\left[ \begin{matrix} 3\times3, 64 \\ 3\times3, 64 \end{matrix} \right]\times3$ | $\left[ \begin{matrix} 1\times1, 64 \\ 3\times3, 64 \\ 1\times1, 256 \end{matrix} \right]\times3$ |
| conv3_x | 64x64 | $\left[ \begin{matrix} 3\times3, 128 \\ 3\times3, 128 \end{matrix} \right]\times2$ | $\left[ \begin{matrix} 3\times3, 128 \\ 3\times3, 128 \end{matrix} \right]\times4$ | $\left[ \begin{matrix} 1\times1, 128 \\ 3\times3, 128 \\ 1\times1, 512 \end{matrix} \right]\times4$ |
| conv4_x | 32x32 | $\left[ \begin{matrix} 3\times3, 256 \\ 3\times3, 256 \end{matrix} \right]\times2$ | $\left[ \begin{matrix} 3\times3, 256 \\ 3\times3, 256 \end{matrix} \right]\times6$ | $\left[ \begin{matrix} 1\times1, 256 \\ 3\times3, 256 \\ 1\times1, 1024 \end{matrix} \right]\times23$ |
| conv5_x | 16x16 | $\left[ \begin{matrix} 3\times3, 512 \\ 3\times3, 512 \end{matrix} \right]\times2$ | $\left[ \begin{matrix} 3\times3, 512 \\ 3\times3, 512 \end{matrix} \right]\times3$ | $\left[ \begin{matrix} 1\times1, 512 \\ 3\times3, 512 \\ 1\times1, 2048 \end{matrix} \right]\times3$ |
| softmax | 1x1 | Average pool, 1000-d fc | | |

The training process involves initializing the model parameters with the following predefined values:

1. Model from Figure 5
2. Specify 16 batch size
3. Specify 200 epoch
4. Specify the activation functions, namely the Sigmoid and Softmax activation functions.

- Evaluation: The model is evaluated using the Binary Cross Entropy loss function, with the following equation:

$$loss\left( o,t \right)=\left\{ \begin{matrix} log(o) & t=1 \\ -log(1-o) & t=0 \end{matrix} \right.$$

where *t* represents the ground truth.

*o* represents the model's output.
